# Self-clustered GAN for precipitation nowcasting

**DOI:** 10.1038/s41598-024-60253-w

**Published:** 2024-04-29

**Authors:** Sojung An, Tae-Jin Oh, Sang-Wook Kim, Jason J. Jung

**Affiliations:** 1 Korea Institute of Atmospheric Prediction Systems, Seoul, 07071 Republic of Korea; 2 CJ Cheiljedang, Seoul, 04637 Republic of Korea; 3 Chung-Ang University, Seoul, 06974 Republic of Korea

**Keywords:** Precipitation nowcasting, Representation learning, Self-supervised learning, Natural hazards, Environmental sciences

## Abstract

This paper proposes a novel GAN framework with self-clustering approach for precipitation nowcasting (ClusterCast). Previous studies have primarily captured the motion vector using only a single latent space, making the models difficult to adapt to disparate space-time distribution of precipitation. Environmental factors (e.g., regional characteristics and precipitation scale) have an impact on precipitation systems and can cause non-stationary distribution. To tackle this problem, our key idea is to train a generator network to predict future radar frames by learning a sub-network that automatically labels precipitation types from a generative model. The training process consists of (i) clustering the hierarchical features derived from the generator stem using a sub-network and (ii) predicting future radar frames according to the self-supervised labels, enabling heterogeneous latent representation. Additionally, we attempt an ensemble forecast that prescribes random perturbations to improve performance. With the flexibility of representation learning, ClusterCast enables the model to learn precipitation distribution more accurately. Results indicate that our method generates non-blurry future frames by preventing mode collapse, and the proposed method demonstrates robustness across various precipitation scenarios. Extensive experiments demonstrate that our method outperforms four benchmarks on a 2-h prediction basis with a mean squared error (MSE) of 8.9% on unseen datasets.

## Introduction

Deep learning has acted as a breakthrough achievement and serves as a turning point in precipitation nowcasting^1–3^. Unfortunately, precipitation nowcasting models utilizing deep neural networks are adversely affected by data blurring problems with increasing forecast lead time. Several studies have highlighted the potential of GANs in effectively tackling blurring issue and demonstrating reliable predictive capabilities. For example, Jing^4^ introduced AENN, which is a network based on GAN that predicts precipitation in 90 min and uses previous radar reflectivity data to overcome blurry prediction. Following the AENN, DGMR^1^ proposed a novel nowcasting system building hierarchical ConvGRU cells as a generator, with two discriminators designed to capture space-time patterns. At another point, researchers utilized diffusion^5,6^, which has the advantage of being free from discriminators and has shown promising results in various real-world applications^7^.

While researchers explore innovative approaches to precipitation nowcasting, deep learning-based models that use deep learning have focused on learning a single latent representation of the precipitation. Approximating rainfall with a single latent space may be overly restrictive, impeding the effective learning of precipitation features. Weather events with different types of precipitation have different characteristics. Convective thunderstorms, drizzles, and many other types of precipitation differ in terms of their spatial patterns, precipitation intensity, lifetime, and moving speed. Chaotic dynamics cause non-stationary precipitation patterns, thereby defining rainfall with a single distribution can be a cause of performance degradation^8–11^. For these reasons, learning representations between similar and dissimilar pairs of precipitation types is crucial for achieving strong performance in precipitation nowcasting. However, precipitation data are inherently high-dimensional and complex, posing difficulties even for domain experts to directly label time-series datasets. Hence, we propose the ClusterCast framework, which utilizes a self-clustering approach for the forecasting task. An SSL-driven clustering methodology facilitates automated labeling of precipitation types within unannotated precipitation datasets, thereby facilitating precipitation prediction according to precipitation types. This approach offers the advantage of seamlessly integrating the clustering and forecasting tasks into a unified module, mitigating potential conflict in representations from each task and fostering adaptable representation learning.

For perspective deep learning architecture, GAN methods often suffer from mode collapse, wherein the generator network learns how to generate plausible outputs but fails to capture various precipitation scenarios. Recognizing the issue of collapsing, many researchers have chosen to cluster GAN architectures, typically as generators or discriminators^12–14^ based on self-supervised learning (SSL). SSL, a type of unsupervised learning that has become popular in computer vision and natural language processing^15^, learns feature representations from the data itself. SSL enhances effective representations from unlabeled data for downstream task through self-generated signals. Diverse image generation via self-supervised GANs^16–19^ enabled robust performance against mode collapse. For example, Sage^16^ introduced a framework utilizing synthetic labels obtained through clustering to mitigate and prevent mode collapse. This approach promotes the disentanglement of variations within and across different classes, thereby facilitating the generation of diverse and realistic labels. These useful applications motivated us to propose a precipitation nowcasting model for learning heterogeneous representations of precipitation by SSL scheme as sub-network of GAN.

To achieve this goal, we leverage the idea of a self-clustering approach of SSL, which formulates time series as disparate latent spaces and exploits such prior knowledge to learn time-series representation. Specifically, ClusterCast leverages heterogeneous latent spaces according to various precipitation types learned by hierarchical resolutions from the sub-network of the generator. ClusterCast efficiently learns non-stationary patterns mitigating the problem of mode collapse and robust various precipitation scenarios. It also learns more powerful representation by leveraging learnable a sub-network for clustering precipitation types which enables stable network interactions. Experiments conducted on real-world datasets demonstrate that ClusterCast achieves over 3% and 8.9% improvement in critical success index (CSI) and MSE compared to four popular benchmark models in precipitation nowcasting, respectively.

In summary, the main contributions of our paper include: (i) We propose a self-clustered generator model to capture the high-dimensional distribution of disparate precipitation types (e.g., drizzles and convective rain), solving the collapsing problem of GAN. (ii) We investigate different self-clustering approaches with a GAN to explore the most suitable and stable method for precipitation nowcasting. (iii) We further enhance our model using an ensemble forecast that samples the uncertainty when radar measures the reflectivity or what may occur as the atmosphere evolves. Notably, we aim to address the following two main research questions; *RQ1:* Compared to the previous time-series nowcasting methods, what is the performance of ClusterCast? *RQ2:* Can we generate future radar frames against precipitation scenarios for unlabeled precipitation data?

## Related works

### Deep generative model for time-series precipitation nowcasting

Precipitation nowcasting is a research-intensive field, especially with the increase of deep learning frameworks for prediction such as models based on ConvLSTM^20^. Traditional precipitation nowcasting consists of a system that predicts future time steps based on an optical flow algorithm^21^ which predicts precipitation evolution by movement extrapolation. Optical-flow-based systems have a limitation in predicting non-linear precipitation patterns as it does not consider the underlying moist physics such as evaporation, condensation, and so on. Deep learning models have recently surpassed optical flow-based weather prediction systems in performance, leading to feasible real-world applications. Shi^20^ were able to effectively predict the space-time evolution patterns of precipitation by combining convolution with RNN^22,23^. However, the algorithm has a limited ability to represent complex movements and the rotation of clouds. Precipitation nowcasting studies have attempted to overcome this shortcoming by constructing hierarchical ConvRNN cells^1,20,22^. For instance, TrajGRU^22^ formulated a loss function according to the precipitation threshold and incorporated hierarchically nested convolution and ConvGRU cells with the optical flow. Other researchers used a combination of convolution and LSTM encoder and adopted multi-resolution connections^24,25^. Sønderby^26^ proposed Metnet, which was used to predict precipitation for the next 8 h by synthesizing observation data. Espeholt^27^ proposed a follow-up model, which expanded its prediction time to 12 h by additionally utilizing numerical weather prediction model output and showed promising short-term forecast results. Most previous studies designed RNN cells, which resulted in blurred images as the forecast time increased. However, predicting fine-scale details is an important element for successful precipitation forecasts. Several groups have focused on developing nowcasting models that preserve the resolution over time. Jing^4^ designed a GAN model with a ConvLSTM generator and two discriminators for radar extrapolation. This model adopted the loss function of the sum of the MSE and mean absolute error (MAE) for the generator and binary cross entropy (BCE) loss for the discriminators. They clipped the radar reflectivity between 0 and 75 decibels and then predicted high-resolution radar data for the next 1.5 h. Ravuri^1^ proposed another GAN-based precipitation prediction model, DGMR, using ConvGRU generators and two discriminators for discriminating spatial and temporal patterns. Not only did Ravuri^1^ successfully develop high-resolution predictable models using only radar observations, but their models delivered performance better than other models when evaluated on the basis of the CSI indicators. Their algorithm targets heavy precipitation, consisting of hinge loss in the discriminator, hinge loss, and MAE in the generator. Recently, there have been efforts to utilize diffusion models to tackle mode collapse in GANs. However, despite these advancements, there remains a risk that generative models may exhibit deviations from physical behaviors, such as generating plausible noise or overlooking domain-specific expertise^6,28^. Therefore, when employing generative models, it is imperative to ensure that the generated samples from the learned distribution adhere to physical realizable.

### Mapping training to solve the mode collapse problem

There were many studies attempting to generate high-resolution images using GANs in the field of computer vision, but the GAN instability issue still remains. The generator attempts to identify one output that seems most plausible to the discriminator, but each iteration of the generator is over-optimized for a particular discriminator, and the discriminator undergoes mode collapse; that is, the model state is trapped in a local minimum of the loss. The unrolled GAN^29^ attempted to solve the mode collapse problem by providing additional information on the discriminator response. VEEGAN^30^ recovers latent distributions to reverse the action of the generator by mapping the data to noise. Diverse image generation via self-supervised GANs^16–19^ enabled robust performance against mode collapse. Lučić^18^ introduced a generative model clustered for unlabeled images based on self- and semi-supervised learning. Sage^16^ suggested clustered GAN training on features obtained via unsupervised feature learning methods for multimodal data. To prevent the generator from generating similar samples, they set the condition of the discriminator and the process of categorizing the image as real or fake^17^. Liu^19^ proposed conditional GAN, which improves image diversity by employing a generator with labels automatically derived from clustering in the feature space of the discriminator. They solved the problem of matching the original labels with newly clustered labels using Hungarian matching. These methods are similar to those used in our work. Our goal is to generate non-blurry future radar frames from light rain to heavy rain by devising a more efficient self-supervised scheme within a unified model. We attempt to approach time-series precipitation nowcasting through a unified framework, employing self-clustering with a GAN. This approach aims to improve the model’s ability to learn better representations by exposing the AI system to precipitation uncertainty.

**Figure 1:**
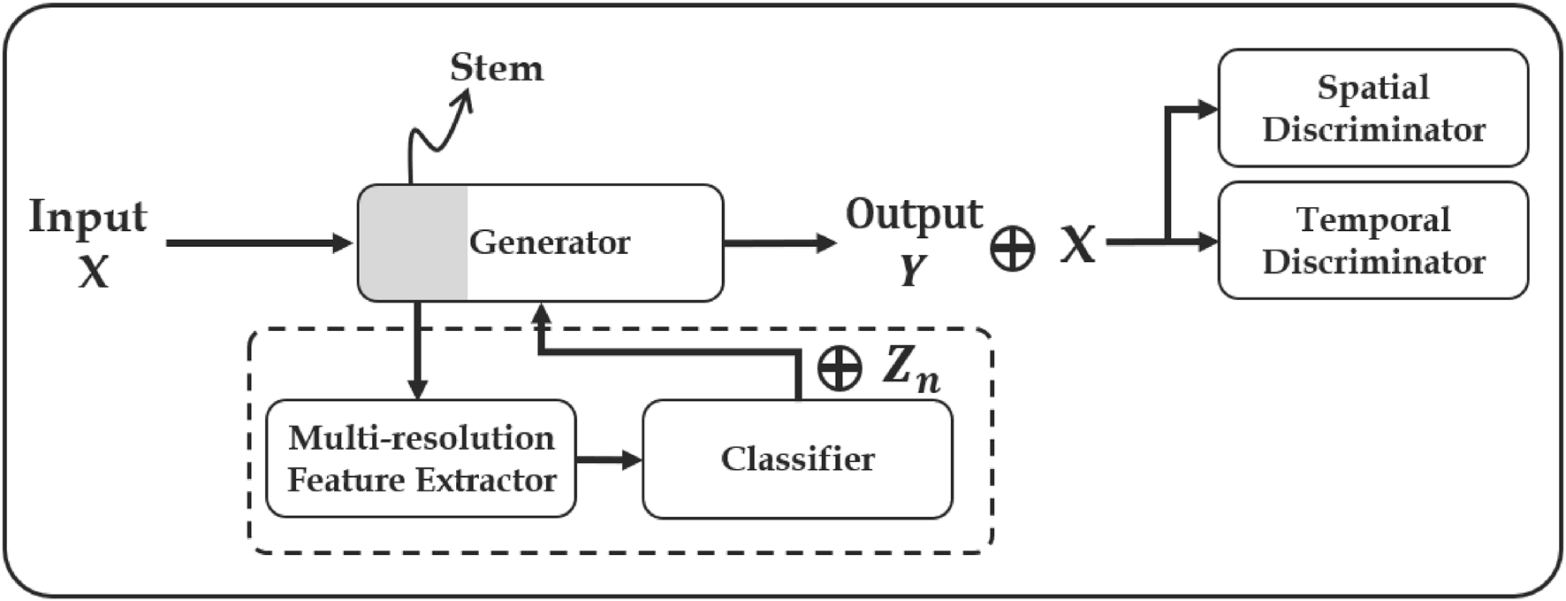
Process flow where the generator predicts radar frames using self-supervised labels. After passing through the stem of the generator the four resolution representations, consisting of a feature extractor containing the convolution layers for each resolution, are fused. The features classify precipitation types from the classifier (section “Clustering methods for self-supervised learning”) and initialize ConvGRU cells with the latent vector according to the type. The specific structure of the network is described in Fig. 3.

**Figure 2:**
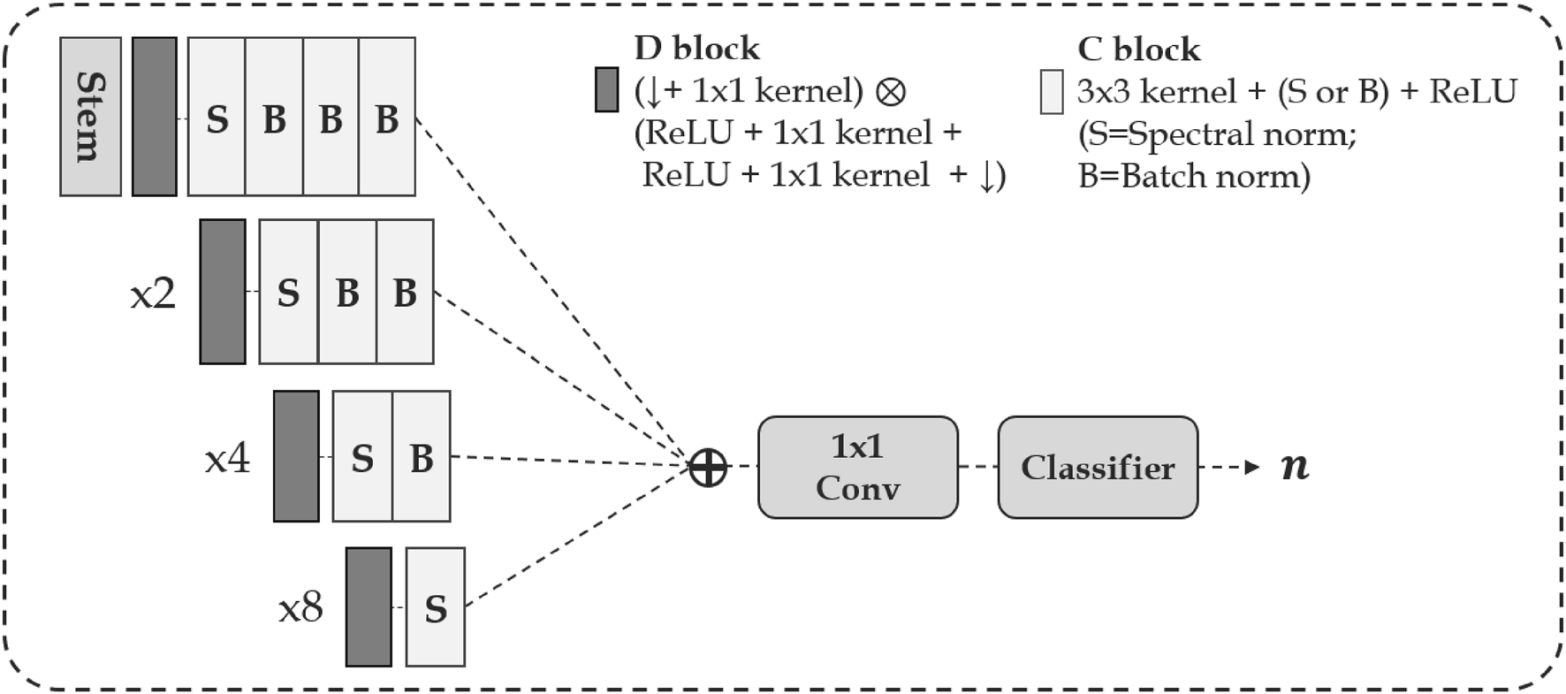
Feature extractor architecture for self-cluster learning. A detailed list of modules in each block is configured as shown on the top right, and ($\downarrow$) represents down-sampling.

## Self-supervised precipitation nowcasting framework

In this paper, we present the architecture of the developed self-clustered generator $\textbf{G}_{\Theta }$. To achieve precipitation nowcasting based on SSL, our approach has two key steps:

- Figure 1: time-series forecasting framework utilizing a self-clustering approach. The generator is structured with hierarchical ConvGRU cells, while the discriminators comprise spatial and temporal components to capture space-time patterns of precipitation.
- Figure 2: sub-network framework combines multi-resolution features to facilitate the learning of both fine-grained local and coarse-grained global interactions. we utilize traditional clustering techniques to provide condition information to the generator through self-clustering.

**Figure 3:**
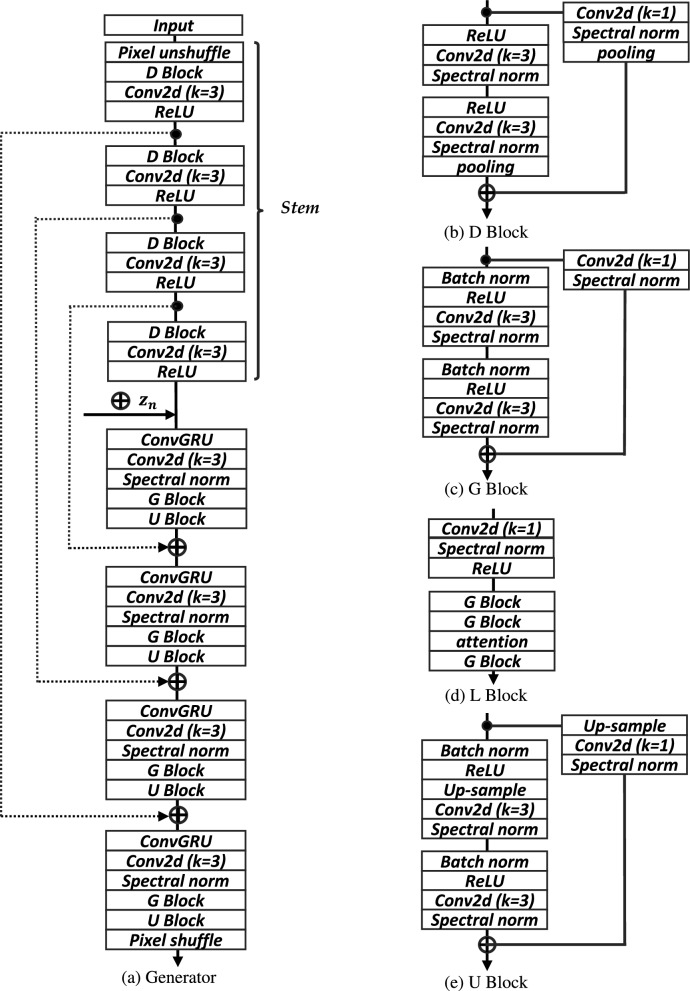
Generator architecture details. (**a**) Generator: clustering precipitation types based on the features of each resolution through an input down-sampling process, referred to as the stem. The generator initializes the distribution $z_n$ according to the clustered results to generate rainfall time-series data that adheres to the distribution of $z_n$. (**b**–**e**) blocks: detailed structures of the generator. The nearest mode is adopted for up-sampling, and attention refers to multi-head attention.

### Self-clustered generator

For a given radar input frames $X=\{x_1, \ldots , x_{i}\} \in \mathbb {R}^{i\times h\times w}$, we generate future radar frames set $Y=\{x_{i+1}, \ldots , x_{i+j}\}\in \mathbb {R}^{j\times h\times w}$ through a self-clustered generator $\text {G}_{\Theta }: X \rightarrow Y$. $\Theta$ is composed of $\theta$ and $\pi$, where $\theta$ represents generator network and $\pi$ refers to sub-network for self-clustering. We denote the underlying latent states according to precipitation type *n* by $z_n \in \mathbb {R}^{d \times \frac{h}{32}\times \frac{w}{32}}$, where *d*, *h*, and *w* are dimensions of latent vectors, height, and weight, respectively. Our framework aims to derive a model for generating radar frames by $\text {G}_{\Theta }(X; z_n)$ that is approximated to the radar frames of cluster n for $z\sim N (0, 1)$, which is a self-clustered among the sampled points from the Gaussian distribution. For learning the temporal and spatial distribution, the model consists of two discriminators, spatial and temporal discriminators $\mathbf {S_{\mu }}$ and $\mathbf {T_{\phi }}$ respectively. This approach reduces the problem of solving the optimization task. The objective function of the self-clustered generator is defined as follows:

1 $$\begin{aligned} \begin{aligned} L_{\mathbf {G_\theta }}&= \mathbb {E}_{X \sim P_{n}}[\mathbb {E}_{z_n} [S(G_{\Theta }(z_n; X))] + T({X; G_{\Theta }(z_n; X)})]-\alpha L_{gauge} + L_{pos}, \\ L_{gauge}&= \frac{1}{hwt} || (\mathbb {E}_{z_n} [G_\Theta (z_n;X)] - Y) \odot w_Y ||, \\ \end{aligned} \end{aligned}$$

where *hwt* represents the mean value calculated across the dimensions of height, width, and lead time. $\alpha$ denotes the hyperparameter used to adjust the ratio of errors for different loss functions, and $\odot$ represents the element-wise multiplication. $L_{gauge}$ represents the weighting $w_Y$ of each pixel based on rainfall intensity, facilitating the learning process for heavy rainfall gauges in sparse precipitation datasets. Given that precipitation data primarily consists of pixels with values under 10 mm, accurately predicting intense precipitation presents a challenge when utilizing the MAE loss function. $L_{gauge}$ can enhance accuracy for heavy rainfall by applying the MAE loss function with weighting. The loss function $L_{pos}$ minimizes positional information using the Dice loss, as outlined below:

2 $$\begin{aligned} \begin{aligned} L_{pos}= & {} \sum _{\forall m \in M} w_m \left( 1 - \frac{2 \cdot |\hat{Y}_m \cap Y_m|}{|\hat{Y}_m| + |Y_m|}\right) . \end{aligned} \end{aligned}$$

here, *M* denotes the set of rainfall thresholds, and $\hat{Y}_m$ represents a conditional matrix derived from $G_{\Theta }(z_n; X)$, where a value is 1 if value exceeds threshold *m*, and 0 otherwise. For same reasons as $L_{gauge}$, $w_m$ serves as a loss weight of $L_{pos}$ based on the rainfall. As a result of the penalty loss output, the position adjusts towards the target pixels in non-overlapping scenarios.

Inspired by self-supervised GAN frameworks^18,19^, ClusterCast learns time-series representations by minimizing the heterogeneous latent spaces according to precipitation types. The approach entails designing a sub-network, denoted as $\text {G}_{\pi }$, which classifies precipitation types by utilizing hierarchical resolution features obtained from historical observations. For achieving self-supervised nowcasting, the input frames undergo four down-sampling process in the stem of the generator. The input frames are transformed into $\chi _{n \in (1, 4)} \in \mathbb {R}^{{2^n}c \times \frac{h}{2^{n}}\times \frac{w}{2^{n}}}$ by down-sampling process. The features at each of the four resolutions, as shown in Fig. 2, are concatenated and processed through convolution layers to classify the precipitation types *n*. Note that such hierarchical resolution features enables the analysis of local to global context of precipitation. After classifying the type, we initialize Gaussian distributions for each component according to the precipitation type. By doing this, our framework helps alleviate the issue of collapsing caused by variations in distributions among different types of precipitation. The initialized latent vector, denoted as $z_n$, passes through the *L* block, which improves performance by rescaling its output probabilities^31^. Subsequently, the latent states $z_n$, along with each resolution features $\chi _{i}$, are inputted into hierarchical ConvGRU cells as follows:

3 $$\begin{aligned} \begin{aligned} h_1&= z_n, \\ u_t&= \phi (\chi _i \oplus h_{t-1},\ w_u),\\ r_t&= \phi (\chi _i \oplus h_{t-1},\ w_r), \\ c_t&= \phi (\chi _i \oplus (\sigma (r_t) \odot h_{t-1}),\ w_c), \\ h_t&= \sigma (u_t) \odot h_{t-1} + (1- \sigma (u_t)) \odot R(c_t), \end{aligned} \end{aligned}$$

where *R* refers to the ReLU activation function. The hierarchical architecture gradually decodes with upsampling modules to multiple levels of representations and generates the future output frames *Y* described in Fig. 3. The output frames are sampled by Monte Carlo estimations as six cases, which estimate the log-likelihood gradient of the precipitation distribution and comprise radar sequences^1^. The $G_{\Theta }(X; z_n)$ is jointly optimized for the classifier, and we update the parameters for two tasks, regression ($\theta$) and clustering ($\pi$) simultaneously at each epoch, using the objective function. Solving the regression task through a single, unified model-based self-clustering approach provides greater stability compared to the two-stage models associated with separately classifying precipitation types and predicting precipitation. Moreover, our framework enables comprehensive representation learning across both tasks, and the generator gains more understanding of the distribution of precipitation types.

### Clustering methods for self-supervised learning

We implement the fundamental self-clustering scheme outlined in section “Self-clustered generator”, and design four sub-networks for the clustering task aimed at learning representations in heterogeneous latent spaces. A self-supervised label ***n*** is sampled from the categorical distribution $P_{n}$ which weighs each cluster proportional to its true size in the training set. In this section, we aim to employ traditional clustering techniques, such as k-means clustering, PCA, and linear-based methods, to explore the most stable and efficient sub-networks. The sub-networks not only enhance the performance of the generator by addressing the mode-collapse problem but also provide robust representations of high-dimensional distributions for predicting future frames. The Table 1 shows the objective functions for the following four clustering methods.

**Table 1:** Objective functions for self-clustering framework, which are proposed by using four traditional clustering methods. Notation. Let $\epsilon _n$ be the centroid of cluster $C_n$ and be the centroid closest to *s*. $C_f$ denotes the encoder layer for clustering to the $H_{t}^{(1)}$ and $t_{\ell }$ is the number of output neurons. $C_f$ represents derived from the expression $L_{L}(y_n)$, and $f(\cdot )$ refer to the nonlinear mapping function at the 5 K iteration.

Notes	Equations
$L_{K}(\{ {\pi _n\}}_{n=1}^{k})$	$\mathbb {E}_{n \sim P_{\pi }}\Bigr [\sum _{n=1}^{k}\sum _{s_c\in C_n} {|| s_c - \varepsilon _n ||}_2\Bigr ]$
$L_{PK}(\{ {\pi _n\}}_{n=1}^{k})$	$\mathbb {E}_{n \sim P_{\pi }}\biggr [\mathbb {E}_{x \sim \pi _{n}}\Bigr [{||P_f(s_c))-\varepsilon _{n}||}_2\Bigr ]\biggr ],\quad \text {where } P_f = min_{U V} = {||s_c - UV^T ||}_1$
$L_{L}(y_n)$	$\frac{exp(C_f(\pi ))}{\sum _{n=1}^{k}exp(C_f(\pi ))},\quad \text {where } C_f(\pi ) = f_{\phi }^{d}\biggr (\sum _{\ell =1}^{d-1}R\Bigr (f_{\theta }^{\ell } C_{1}^{(\ell -1)}\Bigr )\biggr )$
$L_{LK}(\{ {\pi _n\}}_{n=1}^{k})$	$\mathbb {E}_{X\sim P_{\pi }}\Bigr [{\sum _{n=1}^{k}\sum _{s_c\in C_f}||C_f(s_c)-\varepsilon _{n}||_{L_2}}\Bigr ]$

- **Method 1** ($L_K$; *K*-means clustering). *K*-means clustering is a popular unsupervised machine learning algorithm used for partitioning a dataset into a predetermined number of clusters. For the first clustering step, we used random centroid initialization k-means++, and for subsequent re-clustering, we initialized the *K*-means algorithm with the means induced by the previous clustering. Given a set of *s* samples passed through the convolution layer of the generator, $\bigl \{s_1, s_2, \ldots , s_t \bigl \} \subset \mathbb {R}^S$, where each sample represents a *S*-dimensional vector and a number *k*, the *K*-means algorithm aims to group these *S* samples into *k* clusters $\bigl \{ {\pi _n\bigl \}}_{n=1}^{k}$. *K*-means clustering is periodically updated by redefining the cluster centers over a metric induced by the current generator features.
- **Method 2** ($L_{PK}$; PCA with *K*-means clustering). PCA is often used to reduce the dimensionality of the dataset by transforming it into a lower-dimensional space while preserving most of the variance. By reducing the number of dimensions, the computational complexity of K-means clustering can be reduced, especially for datasets with a large number of features. $U = (u_1, \ldots , u_c)$ contains the principal directions and $V = (\text {v}_1, \ldots , \text {v}_c)$ contains the principal components. Here, $L_1$ normalization is beneficial for generalizing the samples^32^. The optimization of the feature extraction for each vector using $L_{PK}(\bigl \{ {\pi _n\bigl \}}_{n=1}^{k})$ is only performed during the first epoch to stabilize the latent space.
- **Method 3** ($L_L$; Linear-based clustering). Linear-based clustering methods often implicitly or explicitly perform dimensionality reduction, where the covariance matrices can capture correlations between features. This algorithm is implemented via an *l*-layer encoder network, which resolves nonlinear mapping to enhance data representation. The model is designed to learn the latent space internally by encoding the hierarchical features derived from the generator stem and applying the softmax function. By exponentially increasing the number of samples, the dimensionality of the data can be reduced, which helps to avoid the curse of dimensionality. All the weights of the encoder and a softmax layer simultaneously update periodically at regular intervals.
- **Method 4** ($L_{LK}$; Linear-based *K*-means clustering). In high-dimensional spaces, the Euclidean distance used in K-means clustering may become less meaningful, as the concept of distance becomes less intuitive in high-dimensional spaces (curse of dimensionality). To address this problem, this method extracts the features with an encoder and applies *K*-means clustering based on the features to derive condition labels using the optimization problem in Table 1. It’s important to note that the value of $\delta$ (the coefficient that balances the generator loss and cluster loss) depends on the specific dataset and experiment, and there is no fixed optimal value. To augment the precision and robustness of clustering, the network is subjected to retraining with a loss function that integrates *K*-means losses for evaluation. To preserve the integrity of the encoder structure, it is essential to fine-tune the loss function using cluster loss and *K*-means loss. This approach enhances the accuracy and stability of clustering by retraining the network with an optimized loss function.

**Algorithm 1:**
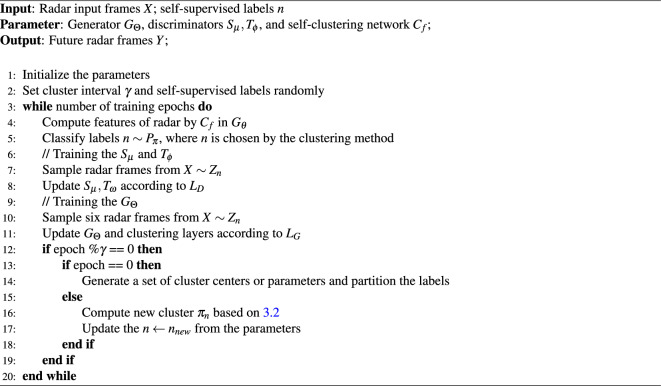
Self-supervised learning with clustering methods for precipitation nowcasting

### Adversarial training

**Figure 4:**
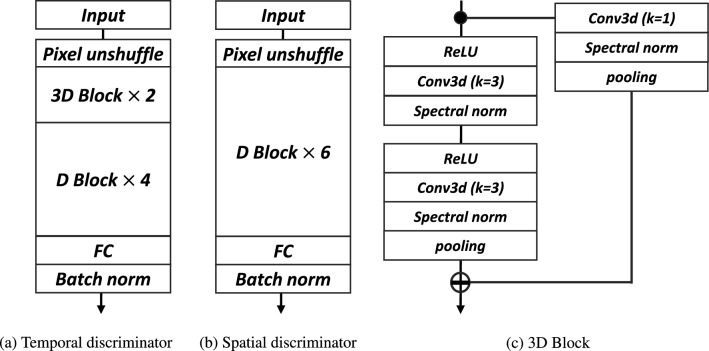
The details of the discriminators. Batch norm, Sectral norm, and FC denote the Batch normalization layers, Spectral normalization layers, and fully connected layers, respectively. (**a**) Temporal discriminator: The network comprises 3D convolution blocks and 2D convolution blocks to learn the temporal distribution of precipitation. (**b**) Spatial discriminator: To capture the spatial distribution, random frames are initially selected. Each frame is then discriminated by six 2D convolution blocks.

The structure of the discriminator is based on that of the DGMR discriminator^1^. Specifically, the discriminator is designed to predict whether the spatio-temporal features in the radar sequence are real or fake. Half of the predicted sequences generated by the generator are randomly selected in the spatial discriminator. The temporal discriminator computes all time-series images cropped to 128 $\times$ 128 pixels. The discriminator consists of that are convolutional and residual layers, each followed by spectral normalization and ReLU activation function. As the input sequence passes through each convolution block, spatial dimension of $S_{\mu }$ decreased by a factor of two and spatial and time dimensions of $T_{\phi }$ decreased by a factor of two at the same time. We address these problems using a novel clustered *Z* using hinge loss, as follows:

4 $$\begin{aligned} \begin{aligned} L_S(\mu )&= \mathbb {E}_{z_n}[R(1-S_\mu (X))+R(1+S_{\mu }(G_{\Theta }(z_n; X)))], \\ L_T(\phi )&= \mathbb {E}_{z_n}[R(1-T_{\phi }(X))+R(1+T_{\phi }({X; G_{\Theta }(z_n; X)}))]. \end{aligned} \end{aligned}$$

here, $L_S(\mu )$ and $L_T(\phi )$ are the spatial and temporal discriminator loss functions, respectively. Spatial and temporal representations were approximated by sampling according to the clustered $z_n$ distribution. $L_S(\mu )$ aims to better preserve precipitation distribution by randomly selecting output frames *Y*. For $L_T(\phi )$, the network discriminates the temporal distribution of Y combined with X by using the Hinge loss function. The discriminators learn to distinguish whether the samples generated by the self-clustered generator are real or fake. Figure 4 shows the network structure of the discriminators.

Algorithm 1 summarizes learning based on the self-supervised learning with GAN. The training ratio of the generator to the discriminators depends on the number of clusters, which is determined based on empirical observations and experimentation, as there is no fixed rule for choosing the optimal ratio. We found that setting a 2:1 ratio for training both the generator and discriminator effectively leads to GAN convergence. However, our model has a limitation: the proposed GAN framework may dominate the training process, potentially resulting in instability depending on the number of clustering labels.

### Ensemble prediction system

Ensemble prediction systems improve prediction skills by addressing uncertainties^33,34^. A simple random perturbation ensemble system was applied to consider the uncertainties from observation errors. The ensemble system consists of 64 members, including the control. After testing under various conditions (not shown), we chose the perturbation members that delivered the best performance, created by multiplying a random number generated from $N(0.95, 0.2^{2})$ by an over 10 mm h^-1^ grid. The ensemble mean forecasts were used as the final result.

## Experiments

This section describes the dataset and experimental setting. To study the effectiveness of the proposed approach for 2-h precipitation prediction, we conducted experiments on four comparison baselines: Rainymotion^35^, ConvLSTM^24^, TrajGRU^22^, and DGMR^1^. The codes for the benchmark models are publicly available on GitHub. The codes represent the official codebase or reproduced implementations. They can be accessed by following the provided hyperlink.

### Dataset

**Figure 5:**
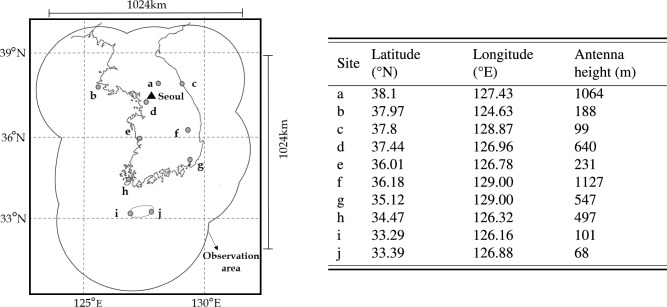
An area of composite weather radar. Composite weather radar in Korea is observed as the S-band, and the radar data approximately covers Korea from 122^∘^ to 132^∘^ longitude and 30^∘^ to 40^∘^ latitude.

South Korea runs 31 weather radars, observed by ministries and synthesized and provided by the Korea Meteorological Administration (https://data.kma.go.kr/cmmn/main.do). Figure 5 depicts the observation area of South Korea. We conducted experiments using the constant altitude plan position indicator (CAPPI), a two-dimensional representation of radar decibel channels at the same altitude. CAPPI reflectivity was provided at a resolution of 500 m, with a size of 2305 (longitude) $\times$ 2881 (latitude) and a temporal resolution of 5 min.

We gathered radar reflectivity data covering a 1024 km^2^ area with a resolution of 500 m. This radar data was then down-scaled to a resolution of 4 km, which was cropped of 256 $\times$ 256 pixels and collected at 10-min intervals. For a 2-h precipitation forecast, we used 6 input frames and generated 12 output frames. Our dataset spans a period of 10 years, from 2012 to the summer of 2021, with the training dataset comprising data from 2012 to 2019, the verification dataset from 2020, and the test dataset from 2021. In total, our dataset contains 132,480 radar data points collected every summer (June–August) between 2012 and 2021 in South Korea. Given Korea’s high annual precipitation levels, with more than 50% occurring during the summer monsoon season known as “Changma,” this dataset is particularly valuable for studying rainfall patterns.

### Rainfall estimation

For the training, we first estimate rainfall intensity by using Z–R relationship. The Z–R relationship is a crucial step in radar-based quantitative precipitation estimation that involves converting reflectivity values into rainfall intensity while considering the types of echoes. Reflectivity is measured in dBZ, and a negative value indicates the detection of very small hydrometeors. To preserve the negative reflectivity value’s meaning, we trained the model by converting reflectivity to rainfall. The reflectivity is converted by the Z–R relationship between the radar reflectivity factor $Z (mm^6 m^{-3})$ and rain rate $R (mm\ h^{-1})$ as follows: $Z = aR^b$, where a and b are parameters obtained empirically depending on the precipitation type. To minimize the precipitation estimation error, constants suitable for the Korean climate (a = 148 and b = 1.59) were applied^36^.

### Data sampling

Samples were systematically extracted from various rainfall events to enable the model to recognize patterns across all precipitation intensities, ranging from light showers to heavy rain. Specifically, sequences exhibiting a spatial distribution of more than 3% of precipitation over 3 h were selected, and the first hour of data was used as input, while the remaining 2 h were used as output. The dataset was comprised of approximately 10,000 examples, and the training subset consisted of 7000 sequences with a Stride 2 (20 min). Moreover, to ensure uniformity in the data, rainfall intensities were capped at 96 $mm\ h^{-1}$, and missing values or empty grids were assigned a value of − 0.1, thereby precluding their utilization in test score calculations. In this study, we propose a novel generative model for nowcasting.

### Detailed experimental setup

The networks were optimized using the Adam algorithm^37^. For the experiment, the minimum and maximum ranges of each set of rainfall data in ConvLSTM and TrajGRU were manually set to [0, 1) using a min–max scaler. Experiments were conducted using a batch size of 16. To compare the AI models with optical flow, we utilized Rainymotion^21^ from Sun^35^ with default settings, employing Affine transform for computing motion vector. For training ConvLSTM^24^, we applied MSE loss and Structural Similarity Index (SSIM) loss function. We configured a 3 $\times$ 3 kernel with leaky ReLU activation, and three ConvLSTM layers with resolutions of 16, 32, and 64 were tested. To compare the AI models with optical flow, we utilized Rainymotion^21^ from Sun^35^ with default settings, employing Affine transform for computing patterns of apparent motion. For training ConvLSTM^24^, we applied MSE loss and Structural Similarity Index (SSIM) loss function. For the training, we reproduced the hierarchical ConvLSTM cells with resolutions of 16, 32, and 64. For TrajGRU, Leaky ReLU served as the activation function, and the ConvGRU cells comprised three layers with 5 $\times$ 5, 5 $\times$ 5, and 3 $\times$ 3 kernels, as detailed in the referenced paper^22^. Each channel number maintained the same sizes as ConvLSTM to facilitate model performance comparison while fixing the number of parameters. The loss function combined MSE and MAE, incorporating weights ranging from 1 to 30 based on rainfall, as described in the paper. The learning rate and momentum were set to 1e−4 and 0.5, respectively. DGMR^1^ was trained using learning rates of 5e−5 for the generator and 2e−4 for the discriminators, respectively. To address GPU memory limitations, we halved the size of the latent vector from its original value to 384, in consideration of GPU capacity. The computation of $L_{gauge}$ involves assigning weights to each pixel based on rainfall intensity: $w_r(\omega ) = \max (\omega , 24)$. The latent space of the Gaussian distribution was initialized in six dimensions. In the experiment, hinge loss was employed, with the optimizer initialized with $\beta _1 = 0.0$ and $\beta _2 = 0.999$. The generative model combines Hinge loss and MAE loss, with weights ranging from 1 to 24 corresponding to rainfall intensity. The loss weight $\alpha$ was set to 20. To ensure stable training, the training ratio of the generator to discriminator was set at 1:1. For ClusterCast, we follow the settings outlined in^1^, with the following adjustments: The weight of the generator loss function is empirically set to $\alpha = 10$. Denoting the rainfall threshold as $M = \{0, 1, 4\}$, where the classes are divided into three, weights $w_m = \{1, 2, 4\}$ are assigned to $L_{pos}$. The training ratio of the generator to the discriminators is set to 1:1. Clustering is performed using the $C_K$ method with 32 groups, and re-clustering occurs approximately every $\delta = 15$ K iterations.

**Table 2:** Evaluation metrics for comparing the performance. The thresholds are set as 1 and 4  mm which are commonly used. Notation. Let *N* represent the number of pixels, while $f_i$ and $p_i$ denote the true observations and the predicted radar frames, respectively. $MAX_{f}$ represents the maximum rainfall intensity. TP, TN, FP, and FN stand for true positives, true negatives, false positives, and false negatives, respectively, between $f_{i}^{\theta }$ and $p_{i}^{\theta }$. $N_x$ and $N_y$ represent the sums of the *x*-grid and *y*-grid divided by the number of neighboring grid cells, denoted by *r*.

$$\text {MSE} = \frac{1}{N} \sum _{i=1}^{N} (f_i - p_i)^2,$$	
$$\text {PSNR} = 10 \log \left( \frac{{\text {MAX}_{f}}^2}{\text {MSE}} \right) ,$$	
$$\text {CSI} = \frac{\text {TP}}{\text {TP}+\text {FP}+\text {FN}},$$	
$$\text {ETS} = \frac{\text {TP}-\frac{1}{N}(\text {TP}+\text {FP})(\text {TP}+\text {FN})}{\text {TP}+\text {FN}+\text {FP}-\frac{1}{N}(\text {TP}+\text {FP})(\text {TP}+\text {FN})},$$	
$$\text {HSS}= \frac{2(TP\times TN - FN \times FP)}{FN^{2} + FP^{2}+2(TP\times TN) + (TP+TN)(FN+FP)},$$	
$$\text {FSS}_r = 1 - \frac{\text {FBS}_r}{\text {FBS}_r^{\text {ref}}},$$	
$$\quad \text {FBS}_r = \frac{1}{N_{x}N_{y}} \sum _{i=1}^{N_{x}} \sum _{j=1}^{N_y} [f_{(i,j)} - p_{(i,j)}]^2,$$	
$$\quad \text {FBS}_r^{\text {ref}} = \frac{1}{N_{x}N_{y}}\left[ \sum _{i=1}^{N_{x}}\sum _{j=1}^{N_y} f_{(i,j)}^{2} + \sum _{i=1}^{N_{x}} \sum _{j=1}^{N_y} p_{(i,j)}^{2} \right] .$$	

### Evaluating metrics

Evaluation metrics are clearly the most significant criterion in the evaluation of the performance the proposed methods, and depending solely on a single metric to verify models may result in biased models receiving favorable evaluations. Therefore, we conducted a comprehensive analysis of model performance from multiple perspectives using various evaluation metrics, as discussed. There are six metrics for evaluating precipitation prediction models, and all the algorithms are evaluated following six standard metrics: MSE, PSNR, CSI, fractions skill score (FSS), equitable threat score (ETS), and heidke skill score (HSS). The evaluation metrics are defined as the predicted probability score and are defined in Table 2. We employed MSE as a metric to evaluate the overall accuracy of time-series predictions. Additionally, we utilized PSNR to measure the sharpness and fidelity of our forecasts. These metrics offer insights: MSE provides a comprehensive measure of predictive accuracy, while PSNR specifically assesses the visual quality and clarity of the predictions. For a comparative analysis of precipitation prediction performance regarding rainfall intensity, the model verifies models based on CSI, ETS, and HSS, which are established metrics for assessing binary accuracy using thresholds. Note that in binary accuracy evaluation, precipitation rainfall is assessed based on pixel-to-pixel, leading to an observed double penalty for non-blurring models such as GAN-based models. These double-penalty problems frequently occur in high-resolution precipitation predictions, and to solve these problems, the FSS is an indicator of evaluating the performance of the prediction model by expanding the spatial scale.

**Figure 6:**
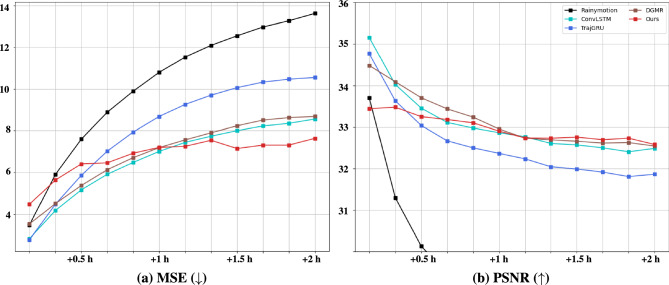
Comparative analysis of lead time performance of benchmarks versus ClusterCast.

## Results

Bearing in mind the formulated RQs, the main goal has been to check if it was able to efficiently generate future radar frames based on precipitation type with unlabeled datasets only through SSL. The following are the main results for comparing performance (see section “Main results”), and the ablation studies were conducted to analyze the clustering results for the unlabeled precipitation dataset (see section “Ablation studies”).

- *RQ1:* Compared to the previous time-series nowcasting methods, what is the performance of ClusterCast? To answer the first question, we conducted a comprehensive analysis of the results using various evaluation metrics. Key findings indicate that the results are i) flexible across a range of precipitation types, and ii) through the learning of distinct distributions for each precipitation type, our model exhibits robustness compared to other models over time.
- *RQ2:* Can we generate future radar frames against precipitation scenarios for unlabeled precipitation data? We attempt to address the second question from the perspective of visualization by considering clustering results based on spatial and temporal variables. Specifically, we mapped high-dimensional features into two and three dimensions by extracting average rainfall and motion vectors (angle and magnitude) to represent and time and spatial characteristics of precipitation. We discovered relationships among the variables in the visualization result and formed meaningful self-supervised labels for these groups. By doing so, we showed the effectiveness of the SSL in nowcasting and provided insight into the clustering scheme.

### Main results

The study compared the results of the proposed algorithm for predicting 2-h precipitation with comparison models. Note that the per-pixel accuracy of the models was evaluated by comparing them after denormalizing using the min–max scaling method for models such as ConvLSTM and TrajGRU. Method 1 (*K*) based on $K=32$, was adopted for SSL as it proved to be the most reliable method, as outlined in section “Ablation studies”. Our brief results are shown in Fig. 6, where ClusterCast outperforms all baselines in most cases. The proposed approach showed the best values for MSE after 60 min of prediction. Although there was no difference from other models in predicting up to 50 min, the proposed method showed a minor increase in loss over the prediction time. In terms of (b) in Table. 3, the proposed method outperformed the previous SOTA approach, achieving a PSNR of 33.906 after 1-h of prediction and 33.580 for the 2-h prediction. Despite slightly lower accuracy than the other models for the initial 50 min, the proposed method outperformed them after this period. The superior results underscore the necessity and effectiveness of using a multi-latent space. GAN-based models might exhibit lower performance in the early time steps of prediction due to the inherent sharpness they strive to achieve, particularly when dealing with real-world radar data or similar sharp data types. This sharpness pursuit can sometimes introduce a slight amount of noise, which may affect the accuracy of early predictions. Another factor that could be speculated to contribute to the initial degradation in results is to distribution shift problem. Time-series precipitation data exhibit non-stationary behavior, where the underlying data distribution changes over time. At the beginning of the forecast horizon, the model might not have fully adapted to these distribution shifts, leading to lower performance. However, despite this initial setback, GAN-based models possess the advantage of learning the temporal distribution effectively over time. This enables them to maintain robust performance as they continue to learn and adapt to the underlying time-series distribution. Note that as the forecast progresses, the model can adjust to these changes and improve its predictions.

**Table 3:** Comparison scores with benchmark models. The 2-h precipitation prediction results are validated using CSI, ETS, HSS, and FSS. The evaluation was conducted at 30-min intervals using thresholds of 1.0 and 4.0 mm. For FSS, we set the radius to 3. Significant values are in bold.

Method	1 mm $\text {h}^{-1}$				4 mm $\text {h}^{-1}$			
	CSI	FSS	ETS	HSS	CSI	FSS	ETS	HSS
(A) Results on the 30 min prediction								
Rainymotion	0.511	0.826	0.478	0.638	0.382	0.736	0.367	0.523
ConvLSTM	0.571	0.826	**0.547**	**0.699**	**0.434**	**0.735**	**0.403**	**0.598**
TrajGRU	**0.572**	**0.840**	0.529	0.679	0.422	0.706	0.396	0.544
DGMR	0.533	0.819	0.507	0.637	0.420	0.705	0.386	0.558
Ours	0.549	0.836	0.520	0.659	0.425	0.727	0.388	0.558
(B) Results on the 60 min prediction								
Rainymotion	0.396	0.697	0.359	0.515	0.265	0.561	0.250	0.383
ConvLSTM	0.410	0.707	0.439	0.597	0.314	0.571	0.303	0.456
TrajGRU	0.468	0.731	0.419	0.588	0.324	0.561	0.289	0.430
DGMR	0.412	0.706	0.422	0.595	0.318	0.567	0.290	0.440
Ours	**0.468**	**0.744**	**0.439**	**0.598**	**0.342**	**0.588**	**0.305**	**0.451**
(C) Results on the 90 min prediction								
Rainymotion	0.329	0.603	0.290	0.435	0.202	0.446	0.187	0.300
ConvLSTM	0.410	0.647	0.369	0.522	0.251	0.468	0.240	0.364
TrajGRU	0.413	0.645	0.356	0.525	0.270	0.473	0.229	0.361
DGMR	0.417	0.644	0.369	0.504	0.260	0.480	0.229	0.365
Ours	**0.426**	**0.673**	**0.376**	**0.526**	**0.278**	**0.486**	**0.245**	**0.372**
(D) Results on the 120 min prediction								
Rainymotion	0.280	0.529	0.241	0.373	0.160	0.361	0.145	0.239
ConvLSTM	0.364	0.583	0.318	0.462	0.202	0.386	0.191	0.297
TrajGRU	0.370	0.587	0.305	0.472	0.223	0.412	0.187	0.310
DGMR	0.372	0.590	0.321	0.467	0.211	0.417	0.195	0.313
Ours	**0.383**	**0.614**	**0.329**	**0.476**	**0.224**	**0.423**	**0.201**	**0.314**

Table 3 represents the performance of each model for two threshold values, 1 and 4 mm. For the 30-min prediction, ConvLSTM performed the best followed by TrajGRU, while Rainymotion showed a similar performance to AI models, indicating little non-linear movement in precipitation during that period. However, after the initial hour, Rainymotion, being an optical flow-based model, showed performance differences compared to other AI models, suggesting gradual changes in precipitation patterns. This implies that AI models may struggle to predict accurately beyond 1 h. Our proposed model demonstrated the best performance in the 2-h precipitation prediction evaluation across all six indices considered, with DGMR ranking second. Regarding GAN-based models, while they do not show performance improvements in rainfall prediction up to 1 h, they exhibited improvements beyond that timeframe. Both models were able to learn the non-linear distribution of rainfall and capture trends effectively as prediction time increased, showcasing strong performance over time.

Regarding the ConvLSTM results, the MSE for the 30-min prediction was the highest, but the model ranked lower for the 2-h prediction. Analysis revealed that over time, there is a noticeable smoothing of the spatial distribution, a gradual decrease in intensity, and an increase in TP. ConvLSTM seems to achieve higher TP due to random chances from numerous rainfall cases. Moreover, as rainfall intensity increases, ConvLSTM’s performance gradually declines, particularly with time, leading to increased model errors. To address this issue, TrajGRU was adopted as a loss function for combining MAE and MSE weighted by rainfall intensity. Additionally, applying the advection scheme improved prediction performance in terms of movement trend and intensity. TrajGRU tends to overestimate over time, leading to increased MSE and significant FN. Despite the higher MSE results, the TrajGRU excels in predicting rainfall intensity. Even when analyzed using ETS, its performance remains excellent from weak to strong precipitation. While TrajGRU’s performance is better in terms of MSE and ETS, it shows strengths in accurate rainfall prediction, suggesting that adjusting weights based on rainfall intensity during training could mitigate overestimations. However, there remains an issue with artifacts introduced by the discretization of the spatial or the time-stepping scheme. The artifact typically refers to any unexpected or undesirable features present in the forecasted data or model output that are not reflective of the underlying patterns or trends in the data.

**Figure 7:**
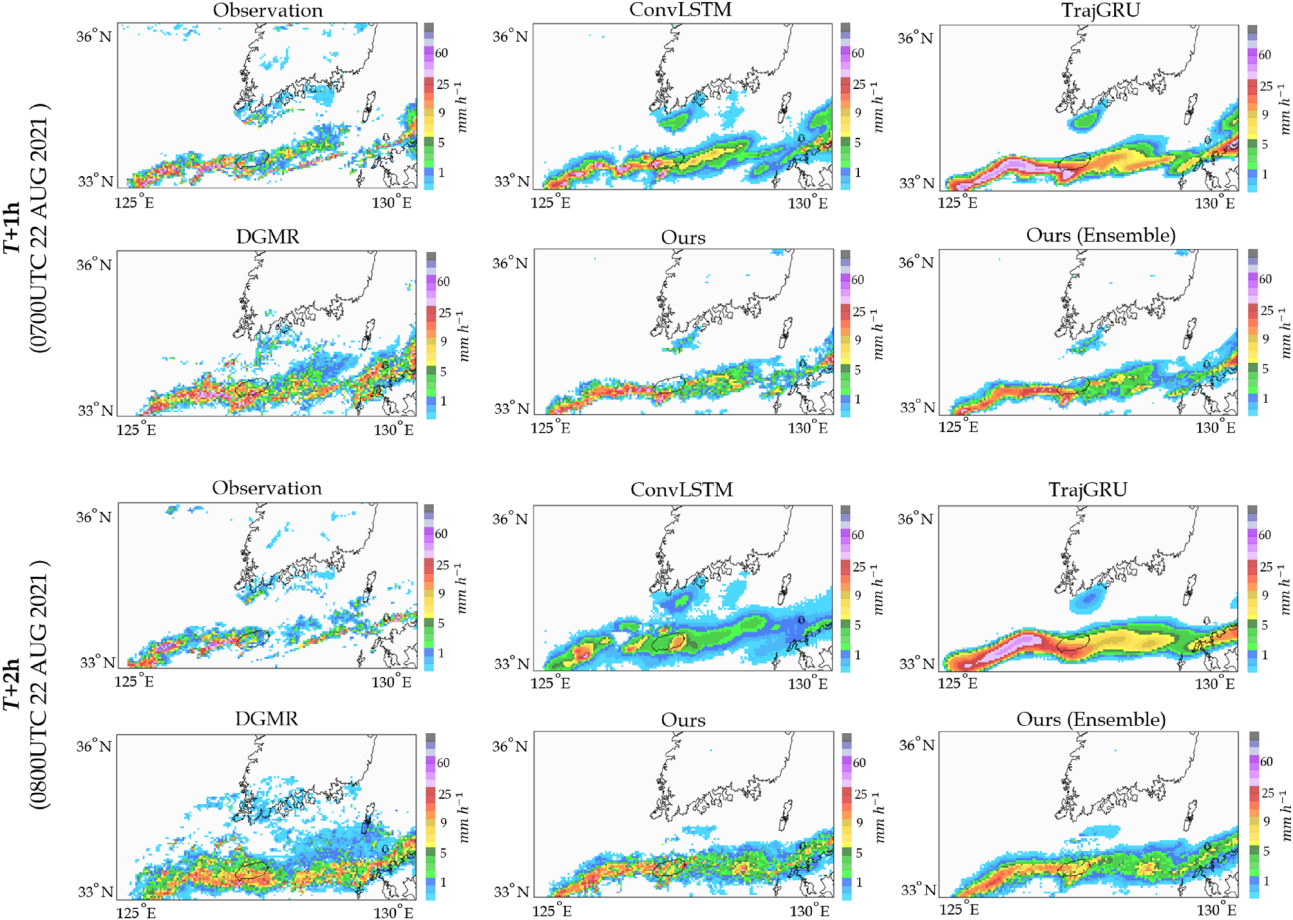
Qualitative comparisons between architectures. The case is a representative type of summer precipitation on the Korean Peninsula; the east Asian monsoon in summer and torrential rain. Based on each model, we visualized the 1-h and 2-h precipitation prediction results, respectively.

Figure 7 highlights the improved precipitation prediction cases generated by our model compared to other models. In particular, our proposed model produces the most accurate simulation of solid elongated precipitation patterns in the monsoon case, where hourly accumulated rainfall exceeds 20 mm. Although the direction of precipitation over the South Sea of Korea differs slightly from the observation, it is worth noting that the direction of precipitation predicted by all deep learning networks remains the same. However, in the case of ConvLSTM and TrajGRU, it is observed that increasing rainfall intensity leads to a smoother spatial distribution, which, in turn, makes it difficult to track the precise location and intensity of precipitation over time. DGMR also exhibits superior simulation capabilities for heavy rain. Nevertheless, the precipitation simulation model requires robustness in accurately capturing a wide range of rainfall intensities, which would help increase CSI scores. To address this issue, we proposed a solution designing heterogeneous latent spaces considering rainfall intensity to enhance the performance of DGMR. The advantage of designing more than one of these latent spaces enhances representation flexibility.

**Figure 8:**
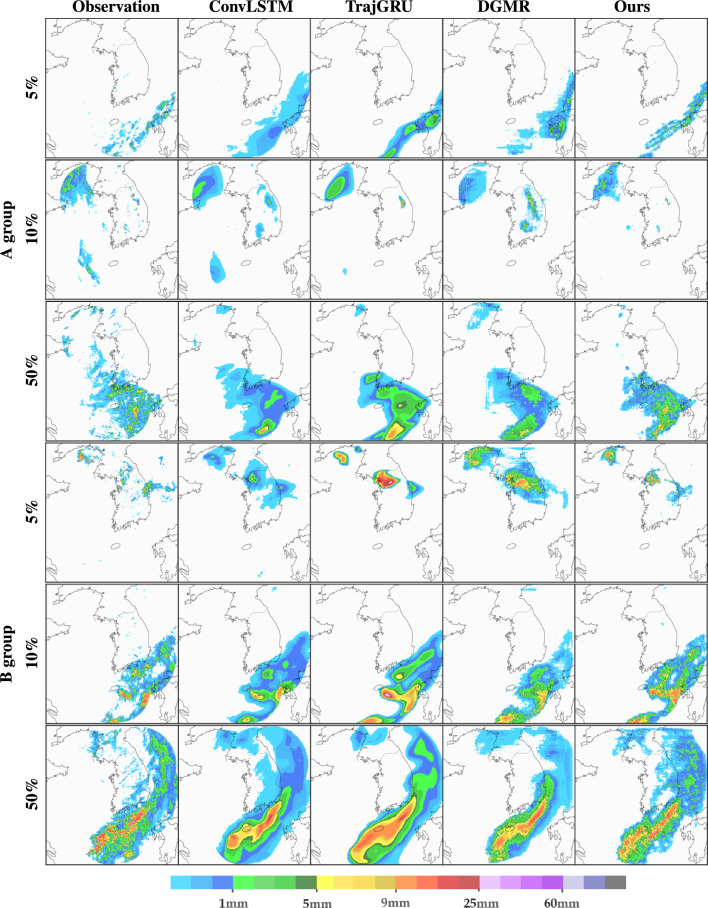
Simulation results of 6 precipitation cases for comparison. The samples are classified according to the precipitation distributions within the test dataset, then randomly selecting those samples. The A group corresponds to cases where the input pixel values indicate precipitation amounts less than 10 mm, while the B group represents cases with 10 mm or exceed rainfall. The simulation of the DGMR and the proposed model is shown in the image to fairly closely approximate the natural radar, which can deliver more specific information. Also, the proposed model best simulates the dangerous weather conditions occurring downtown, and they are quite similar to the ground truth in terms of intensity.

Korean weather has several types of precipitation, including topographical torrential rain, atmospheric instability-induced precipitation, and typhoon-induced nationwide precipitation, which display considerable deviations. In this study, we aimed to visualize the predictions of different precipitation types, which are illustrated in Fig. 8. The figure demonstrated that our method simulated highly plausible images, which closely matched the patterns of the actual rainfall intensity, ranging from light to heavy rainfall.

**Table 4:** Comparison of CSI and MSE scores according to clustering methods. *K*, *P*, and *L* refer to *K*-means clustering, PCA, and a linear-based model, respectively. $L_{pos}$ and *E* denote a model based on a dice loss and ensemble of methods. $(\cdot )$ of $K_{(\cdot )}$ indicates the number of clusters. Significant values are in bold.

Methodology	MSE ($\downarrow$)				CSI 2.0 mm/h ($\uparrow$)			
	+0.5h	+1h	+1.5h	+2h	+0.5h	+1h	+1.5h	+2h
(A) Comparison with baseline study								
DGMR (Baseline)	5.362	7.164	8.221	8.716	0.454	0.373	0.325	0.291
$K_{32}$	5.791	6.772	7.581	8.117	0.482	0.384	0.329	0.298
$K_{32}+L_{pos}$ (Ours)	**4.680**	6.465	7.194	7.552	0.484	0.401	0.347	0.299
$K_{32}+L_{pos}+E$	4.853	**5.638**	**6.402**	**6.936**	**0.502**	**0.422**	**0.366**	**0.317**
(B) Comparison based on the number of clusters								
$K_{16}+L_{pos}$	6.312	7.32	7.847	7.878	0.469	0.357	0.306	0.262
$K_{64}+L_{pos}$	5.429	7.129	7.402	7.982	0.494	0.365	0.324	0.290
(C) Comparison based on clustering methods in section “Clustering methods for self-supervised learning”								
$E_{32}+L_{pos}$	5.591	6.956	7.875	8.498	0.482	0.384	0.329	0.300
$PK_{32}+L_{pos}$	5.309	6.624	6.911	7.210	0.462	0.381	0.328	0.273
$EK_{32}+L_{pos}$	6.453	7.048	7.174	7.902	0.475	0.398	0.349	0.304

### Ablation studies

In order to validate the effectiveness of the clustering components designed for precipitation forecasting, we conducted ablation studies, and the results are presented in Table 4. Specifically, we applied each of the clustering methods proposed in section “Clustering methods for self-supervised learning”, and then experimented with comparing the MSE and CSI using the same dataset. The results indicated that all of the proposed clustering techniques were effective for generating radar frames, which helped to improve the performance of the *K*-means clustering method. Combining an encoder with *K*-means clustering achieved the best performance for heavy rain events. SSL algorithms trained using *K*-means clustering (Method 1) were found to be reliable for predicting all types of precipitation. Conversely, clustering using an encoder layer with softmax led to instability in predicting radar frames.

**Figure 9:**
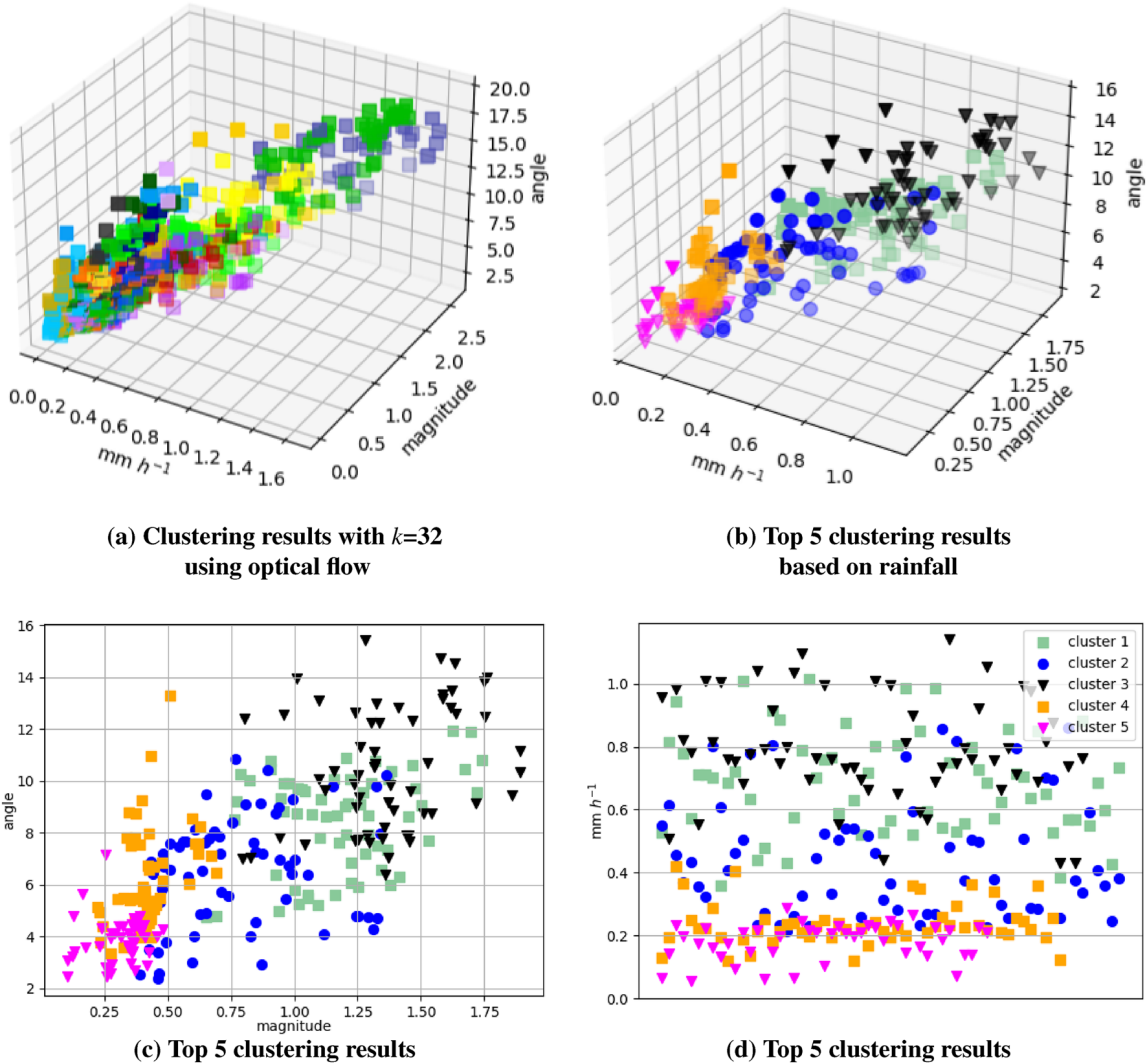
Clustering results of the test dataset using *K*-means clustering. (**a**) Visualization of results grouped into 32 clusters. (**b**) Visualization of predominant findings in the test dataset, representing 7.4%, 7.4%, 6.5%, 6.8%, and 5.3% of the total, respectively. The x-axis represents temporal rainfall variability, while the y-axis depicts mean rainfall. (**c**) Top 5 clustering results by calculating the magnitude and angle of optical flow between the first and last frames of input in order to measure temporal changes. (**d**) Top 5 clustering results based on mean rainfall rainfall variability.

The clustering process was initiated with ten central clusters, which were subsequently partitioned into 16, 32, or 64 new groups. We utilized six radar images as input and applied SSL with various clustering methods. When comparing the clustering results to $K_{32}$ clustering results were found to be the most effective. In experiments with fewer than $K_{16}$, the performance remained similar to that of the existing model. However, previous research indicated that clustering at less than 16 groups decreased performance when combining convolutional layers and *K*-means clustering^38–40^. Although we designed more filters than the referenced paper when clustering into fewer than 16 groups, our model’s performance did not improve on an unseen dataset. On the other hand, clustering using too many input sequences can lead to difficulty in approximating all spaces stably because they map the latter spaces with *K* labels. In this regard, the effectiveness of the forecasting task relies on the number of clusters utilized, which directly influences its parameter configuration. As the number of clusters increases, the instances within each cluster decrease, potentially reducing the training load required for the generator compared to the discriminator. As the number of clusters increases, the instances within each cluster decrease, potentially resulting in representations being less learned in a few latent spaces of the generator compared to the discriminator. Therefore, we adjusted the ratio of generators to discriminators to 1:1, 2:1, and 4:1, accompanied by corresponding adjustments to the learning rates. In our experiments, we explored four different learning rates-1e−4, 1e−5, 5e−4, and 5e−5-aiming to identify the most optimal parameter setup.

We defined the problem of classifying precipitation types as a means to design an adaptive latent space in the generator. For clustering purposes, extracted high-dimensional vectors from the generator stem were visualized by defining two arbitrary variables to represent the clustered results. To capture spatial information, we averaged the rainfall of the input radar frames. For temporal patterns, we calculated optical flow to represent motion and averaged the magnitude and angle of the input radar frames. In Fig. 9, we visualized 32 clustered results and the top 5 clusters with the highest proportions. As shown in the figure, the centroid of the most prevalent cluster had a spatial precipitation of 0.68 mm (including areas with no rainfall), a magnitude of 1.184, and an angle of 8.467. Analyzing the results of this case, it corresponds to a typical occurrence of rainfall during the Korean summer season, characterized by the formation of precipitation-bearing cloud bands over the West Central Sea due to the intensification of warm and moisture-laden southwesterly winds from the south of a low-pressure system^41,42^.

Specifically, the case ranked in the top three represented a coverage of approximately 0.89% or higher for over 10 mm and appeared to correspond to precipitation events at the level of heavy rain with rapid movement. The most severe precipitation scenario recorded a frequency of 3.41% for 10 mm or more, accompanied by an average wind speed of 16 m/s. When we analyze each cluster, these top five types of precipitation commonly occur during the summer in Korea^43,44^. The results indicate that multi-latent space learning of the generator is well-trained from a logical perspective. Additionally, implementing an ensemble approach resulted in a 3.3% reduction in MSE. As nonlinear interactions within the precipitation, even minor changes in the input can result in notable fluctuations in predictions. Leveraging an ensemble technique can bolster the reliability of the prediction model by mitigating uncertainty associated with sensitive precipitation events.

## Conclusion

This paper introduced a novel self-clustered generator for precipitation nowcasting, facilitating heterogeneous representation learning. We hypothesize that nowcasting models approximated with a single Gaussian distribution are a restrictive assumption for predicting all precipitation scenarios. To validate this hypothesis, extensive experiments were designed and conducted to assess the accuracy of predictions. The experimental results demonstrated that our proposed method more accurately time-series forecasting while mitigating mode collapse issues. Our framework enables predicting non-blurry future radar frames, which is robust against diverse precipitation types. In addition, a simple ensemble system was utilized to enhance the performance of our proposed model. We believe that ClusterCast, based on SSL and incorporating various traditional clustering methodologies, will aid readers in designing future time series nowcasting models against different precipitation scenarios.

## Data Availability

The data that support the findings of this study are available from Korea Meteorological Administration but restrictions apply to the availability of these data, which were used under license for the current study, and so are not publicly available. Data are however available from the authors upon reasonable request and with permission of Korea Meteorological Administration.
